# Human Tissue Kallikrein 1 Is Downregulated in Elderly Human Prostates and Possesses Potential In Vitro Antioxidative and Antifibrotic Effects in Rodent Prostates

**DOI:** 10.1155/2021/8877540

**Published:** 2021-04-30

**Authors:** Mengyang Zhang, Changcheng Luo, Dongxu Lin, Kai Cui, Zhong Chen, Jihong Liu

**Affiliations:** ^1^Department of Urology, Tongji Hospital, Tongji Medical College, Huazhong University of Science and Technology, Wuhan, 430030 Hubei, China; ^2^Institute of Urology, Tongji Hospital, Tongji Medical College, Huazhong University of Science and Technology, Wuhan, 430030 Hubei, China

## Abstract

**Objective:**

The aim of the present study was to investigate the protective effects and mechanisms of KLK1 on aging-related prostate alterations and search clues about the application of KLK1 to the treatment of human BPH.

**Methods:**

Thirty-six rats including 26 male wild-type SD rats and 10 transgenic rats were fed to 3- or 18-month-old and divided into three groups: young WTR (yWTR) as the control (*n* = 16), aged WTR (aWTR) (*n* = 10), and aged TGR (aTGR) (*n* = 10). The prostates of the three groups of rats (10 rats per group) were harvested to evaluate the levels of KLK1 expression, oxidative stress, fibrosis, and involved signaling pathways, such as NO/cGMP, COX-2/PTGIS/cAMP, and TGF-*β*1/RhoA/ROCK1, via quantitative PCR, Western blot, histological examinations, and ELISA. Moreover, the remaining 6 yWTRs were sacrificed to obtain primary prostate fibroblast and aortic endothelial cells, and a coculture system was built with the cells for the verification of above signaling pathways *in vitro*. And the direct effects of bradykinin on prostate cells were detected by MTT experiment. Prostate specimens of 47 patients (age from 48 to 92 years) undergoing BPH surgery were collected after approval. Histological examinations and KLK1 IHC were preformed to analyze the relationship between KLK1 expression and age and prostate fibrosis.

**Results:**

The human KLK1 gene only existed and was expressed in aTGR. The prostate of young rats expressed more KLK1 than the aged and the expression of KLK1 in prostate decreased with age in humans (*r* = −0.347, *P* = 0.018). Compared to the aWTR group, the yWTR and aTGR groups showed milder fibrosis, less oxidative stress, upregulated NO/cGMP, and COX-2/PTGIS/cAMP signaling pathways and inhibited TGF-*β*1/RhoA/ROCK1 signaling pathway. In the coculture system, KLK1 suppressed TGF-*β*1-mediated fibroblast-to-myofibroblast transdifferentiation via cleaving LMWK to produce the BK which upregulate eNOS expression and NO production in endothelial cells. BK not only slightly stimulated the proliferation ability of prostatic stromal cells but also upregulated iNOS and inhibited TGF-*β*1 expression in them.

**Conclusion:**

KLK1 protects prostate from oxidative stress and fibrosis via amplified NO/cGMP signal in aged rats. The decrease of KLK1 expression with aging is laying the groundwork for the application of KLK1 to the treatment of human BPH. The current experimental data showed that the side effects of KLK1 on the prostate cell were not obvious.

## 1. Introduction

Benign prostatic hyperplasia (BPH) refers to the proliferation of stromal and/or epithelial layers of the prostate and may cause urodynamic obstruction, even lead to lower urinary tract symptoms (LUTS). Aging is a prerequisite for the development of BPH. The prevalence of BPH rises with age, it is 20% of men at age 40, but increases to 70% at age 60, and in autopsy studies, BPH can be detected in almost 80% of men at age 80 [1]. By the stereological analysis, it has been suggested that BPH is primarily a stromal process, and increased fibrosis has also been implicated in it [2, 3]. The patients with symptomatic BPH have a higher stroma-to-epithelium ratio than asymptomatic patients [3]. Certain patients are unresponsive to conventional treatment for LUTS because of the presence of increased fibrosis within the prostate [4]. Fibrosis in BPH is promoted by the activation of excessive *α*-smooth muscle actin- (*α*-SMA-) expressing myofibroblasts, which are differentiated from fibroblasts and deposit extracellular matrix (ECM) components [5, 6]. Transforming growth factor-*β*1 (TGF-*β*1) can upregulate the activation of the ras homolog family member A (RhoA)/Rho-associated coiled-coil containing protein kinase 1 (ROCK1) pathway to promote cytoskeletal rearrangement in various types of cells, ultimately inducing the pathological process characterized by fibroblast-to-myofibroblast differentiation [7]. Haudek et al. reported that the RhoA/ROCK1 pathway was implicated in cardiac fibrosis [8]. Zhang et al. confirmed the inhibitory effect of ROCK1 deletion on the induction of profibrotic cytokines [9].

Human tissue kallikrein (KLK1) is a serine protease that cleaves low molecular weight kininogen (LMWK) to produce the kinins, especially bradykinin (BK) [10]. BK-related peptides bind to B1 and B2 bradykinin receptors to activate a number of downstream targets such as nitric oxide (NO), cyclic guanosine monophosphate (cGMP), prostacyclin, and cyclic adenosine monophosphate (cAMP) [11]. Kininogens, kallikrein, kinins, kinin degrading enzymes, and kinin receptors constitute the kallikrein-kinin system (KKS) [12]. In various experimental animal disease models, stimulating the KKS through overexpression of KLK1 or other methods has been demonstrated to be beneficial to many diseases through its anti-inflammatory, antifibrotic, and antioxidative actions [11, 13].

In recent years, NO/cGMP pathway in the prostate has been an important area of study. NO exerts its effects by activating soluble guanylyl cyclase which in turn leads to the formation of cGMP and multiple effects including relaxing prostate smooth muscle, leading to the weakness of dynamic component in bladder outlet obstruction [14]. Meanwhile, amplified NO/cGMP pathway increases the relaxation of vascular smooth muscle, leading to increase in tissue oxygenation in the prostate [15]. In addition, NO attenuates TGF-*β*1-induced myofibroblast differentiation of prostatic fibroblasts which plays a key role in BPH development and prostate fibrosis, via its antagonistic effect to NADPH oxidase 4 (NOX4) and reactive oxygen species (ROS) downstream of TGF-*β*1 [5]. In vitro experiments show that stimulating generation of intracellular cGMP by the soluble NO donor sodium nitroprusside (SNP) and pharmacological inhibition of phosphodiesterase 5 (PDE5) suppress TGF-*β*1-mediated fibroblast-to-myofibroblast transdifferentiation [5, 16]. In endothelial cells, NO is synthesized mainly from L-arginine by endothelial NO synthase (eNOS), while asymmetric dimethylarginine (ADMA) is a powerful inhibitor of eNOS through competition with L-arginine to bind to the active site [17]. Dimethylarginine dimethylaminohydrolase 2 (DDAH2), a hydrolase prevalent in endothelium, can degrade ADMA into citrulline and dimethylamine and increase NOS activity [17]. Our previous study found that in the penile tissue of human KLK1 transgenic aged rats, the expression of DDAH1 and DDAH2 was higher than that of wild-type aged rats, and the content of ADMA was lower than that of wild-type aged rats [18]. Besides the NO/cGMP pathway, cyclooxygenase 2 (COX-2)/prostaglandin I2 synthase (PTGIS)/cAMP pathway may also participate in the attenuation of fibrosis by KLK1. Rodriguez found BK could induce protein expression and enzymatical activation of COX-2 [19]. Gallagher proved BK reduced collagen gene expression through enhanced prostacyclin production [20]. COX-2 and PTGIS are the key enzymes in endothelial cells to produce prostaglandin E which can enter the smooth muscle cells to increase the cAMP level and induce the relaxation of them [18].

Our previous work suggested human KLK1 may preserve erectile function in aged rats via activation of the NO/cGMP pathway and reduce corporal fibrosis via regulation of TGF-*β*1-related signaling pathways [21, 22]. And in this past research, accidentally, we found an interesting phenomenon that the prostates in aged transgenic rats harboring the human KLK1 gene looked “younger” than that in aged wild-type rats. This suggested that KLK1 might become a novel potential target against BPH. Therefore, we conducted this animal experiment to explore the specific mechanism of the protective effect of KLK1 on aging-related changes in rat prostates. Meanwhile, we primarily isolated and cocultured the endothelial cells and prostatic fibroblast of rats, to determine the potential signaling pathway of KLK1 against TGF-*β*1-induced myofibroblast differentiation *in vitro*. On the other hand, although the benefits of KLK1 have been studied in many animal experiments, the application of KLK1 in human is still very limited. Therefore, we used surgical specimens of patients with BPH to study the correlation between KLK1 expression and age, laying the groundwork for the application of KLK1 to the treatment of human BPH.

## 2. Material and Methods

### 2.1. Experimental Animals

This study was approved by the Ethics Committee and Academic Administration Committee of Tongji Hospital, Tongji Medical College, Huazhong University of Science and Technology (Wuhan, China). All experiment procedures were in strict accordance with the Guide for the Care and Use of Laboratory Animals published by the US National Institutes of Health. All rats were individually housed in a conventional animal facility with laminar flow, maintained at 20°C ± 1°C and 50% ± 10% relative humidity with a 12 h light/12 h dark photoperiod, and bred by professional breeders. A total of 36 male SD rats were used, 26 of which were wild-type SD rats (WTRs) obtained from the Laboratory Animal Center of Tongji Hospital, and the remaining 10 rats were transgenic rats (TGRs) harboring the human KLK1 gene.

### 2.2. Acquisition of the Transgenic Rat (TGR)

As shown in our previous study [21], we obtained TGRs harboring the human KLK1 gene as a generous gift from the Max Delbrück Center for Molecular Medicine (Berlin, Germany). The production of transgenic rats and selection of homozygous offspring were described previously [23].The TGRs were generated by microinjecting a 5.6-kb DNA fragment containing the entire human KLK1 gene under the control of the heavy metal-responsive mouse metallothionein promoter into the oocytes of Sprague-Dawley (SD) rats. Presence of the transgene in genomic DNA extracted from the tails of 1-week-old rats was verified by Southern blotting. Only healthy 8-week-old male offspring with the homozygous human KLK1 gene were selected for our further experiments.

### 2.3. Experimental Design

**Figure 1:**
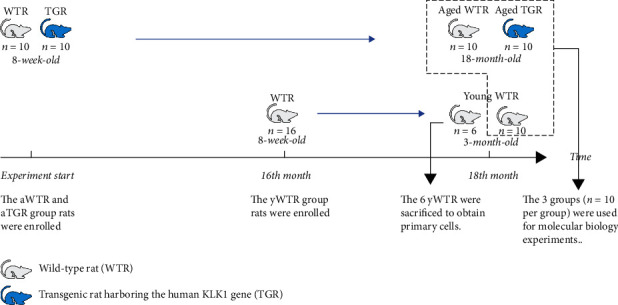
Schematic illustration of experimental design.

Thirty-six rats were divided into three groups: the young WTR group (yWTR) as the control group (3-month-old, *n* = 16), the aged WTR group (aWTR) (18-month-old, *n* = 10), and the aged TGR group (aTGR) (18-month-old, *n* = 10). All the rats (weighing 180-220 g) were bred under the same conditions from 8-week-old until they were 3-month-old and weighed 250-300 g or were 18-month-old and weighed 450-550 g, and then, they were sacrificed at the same time to carry out the follow-up experiments (Figure 1). It should be pointed out that 6 yWTRs were sacrificed to obtain primary cells, and the prostates of other rats were used for molecular biology experiments.

Rats were weighed and then killed with a lethal dose of sodium pentobarbital (180 mg/kg, i.p.). The ventral lobes of prostate glands were quickly removed. One-third of the bilateral ventral lobes were maintained overnight in 4% paraformaldehyde (Beyotime Biotechnology, China) and then embedded in paraffin for histologic studies. The remaining prostate tissues were harvested and stored at -80°C for other experiments.

### 2.4. Verification of TGR

In order to detect the expression of human KLK1 gene in the prostate tissues of rats, we used conventional polymerase chain reaction (PCR) and agarose gel electrophoresis, real-time reverse transcriptase-PCR, and Western blot to determine the human KLK1 in frozen prostate samples at the level of DNA, mRNA, and protein levels, respectively.

### 2.5. Obtaining the Human Prostate Specimens

To lay the groundwork for the future application of KLK1 to the human body, we collected prostate specimens of patients undergoing BPH surgery and conducted a series of background studies. Formalin-fixed paraffin-embedded prostate tissue blocks were obtained from 47 patients who were diagnosed with BPH and underwent transurethral prostatectomy in Tongji Hospital from 2018 to 2019, age from 48 to 92 years. All retrospective clinical data analyses and prostate specimen collection were performed after obtaining informed consent from all patients and approval of the Ethics Committee and Academic Administration Committee of Tongji Hospital.

### 2.6. Cell Culture

#### 2.6.1. Rat Primary Prostatic Fibroblast (RPrPF)

**Figure 2:**
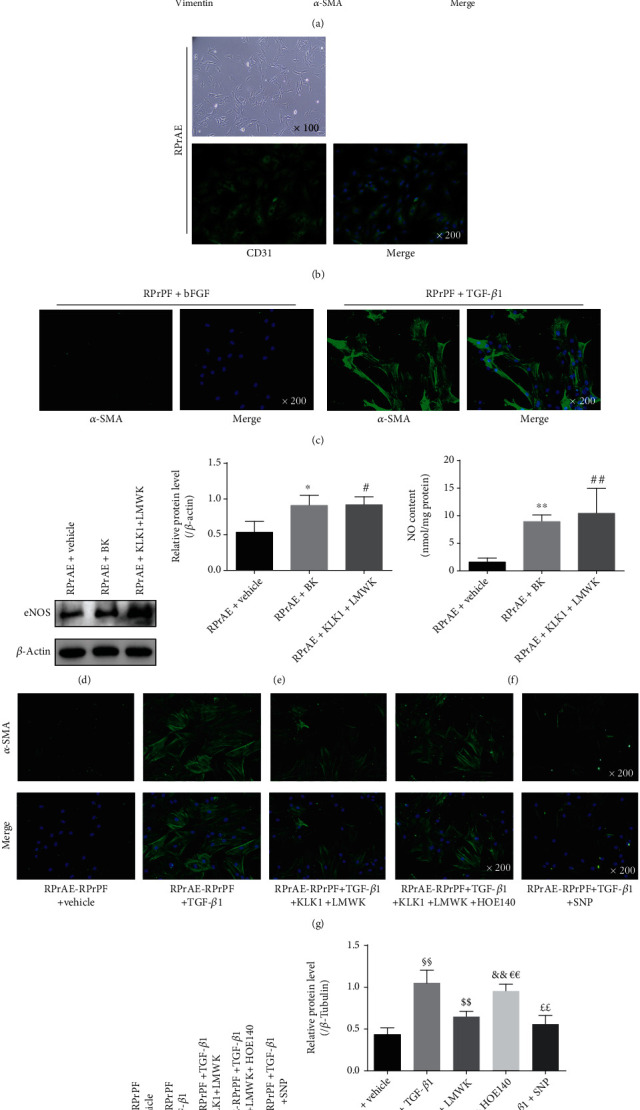
KLK1 could inhibit fibroblast-myofibroblast transdifferentiation induced by TGF-*β*1 in RPrAE-RPrPF coculture system. (a) The morphology of RPrPF (magnification ×100) and the verification of RPrPF through the expression of E-Cadherin (red), vimentin (red), and *α*-SMA (green) (magnification ×200). (b) The morphology of RPrAE (magnification ×100) and the verification of RPrAE through the expression of CD31 (green) (magnification ×200). (c) The fibroblast-myofibroblast transdifferentiation induced by TGF-*β*1 in RPrPF (*α*-SMA, green; magnification ×200). (d, e) Protein expressions of eNOS normalized to *β*-actin in RPrAE under KLK1, LWMK, or BK. (f) NO content in RPrAE under KLK1, LWMK, or BK. (g) Representative IF photos of *α*-SMA (green) expression level changes at the administration of TGF-*β*1, KLK1, LWMK, HOE140, and SNP in RPrAE-RPrPF coculture system. (h, i) Protein expressions of *α*-SMA normalized to *β*-Tubulin in RPrAE-RPrPF coculture system by Western blot. Each bar represents mean ± SD of 3 independent repeated experiment. ^∗^*P* < 0.05, ^∗∗^*P* < 0.01 (RPrAE + BK vs. RPrAE + vehicle). ^#^*P* < 0.05, ^##^*P* < 0.01 (RPrAE + KLK1 + LMWK vs. RPrAE + vehicle). ^§§^*P* < 0.01 (+ TGF-*β*1 vs. + vehicle). ^$$^*P* < 0.01 (+ TGF-*β*1 + KLK1 + LMWK vs. + TGF-*β*1). ^&&^*P* < 0.01 (+ TGF-*β*1 + KLK1 + LMWK + HOE140 vs. + vehicle). ^€€^*P* < 0.01 (+ TGF-*β*1 + KLK1 + LMWK + HOE140 vs. + TGF-*β*1 + KLK1 + LMWK). ^££^*P* < 0.01 (+ TGF-*β*1 + SNP vs. + TGF-*β*1).

As previously reported [24], rat ventral prostate tissue was obtained in sterile environment and minced into 1 mm [3] pieces under nonenzymatic condition, then placed in completed primary fibroblast medium in T25 flask (Corning, USA) as explant cultures. The completed primary fibroblast medium consisted of 88% DMEM medium (HyClone, USA) and 10% fetal bovine serum (Gibco, USA), supplemented with 1% penicillin/streptomycin (HyClone, USA), 1% ITS Liquid Media Supplement (Sigma, USA) and 1 ng/ml basic fibroblast growth factor (bFGF; PeproTech, USA). Medium was replaced every 48 h. Monolayer cultures of fibroblast-appearing cells emanated from peripheral edges of organ explants were allowed to reach confluence. Prostate explants were mechanically removed, and the remaining monolayers were purified by differential adhesion method. Briefly, the cells were digested in 0.05% trypsin-ethylenediaminetetraacetic acid (Gibco, USA), and only the faster adherent fibroblasts were retained for subculture. Primary cultured fibroblastic cells were verified using immunofluorescence (IF) for E-Cadherin, vimentin, and *α*-SMA (Figure 2(a)).

#### 2.6.2. Rat Primary Aortic Endothelial Cells (RPrAE)

As previously reported [25], rat thoracic aorta was collected with the attached tissue clearly removed under sterile condition. These aorta segments were seeded in T25 flask with the endothelium facing down and removed 2 days after endothelial sprouting started. The primary endothelial cell growth medium was commercially available and contained endothelial cell growth supplement, 10% fetal bovine serum, and 1% penicillin/streptomycin (ScienCell, USA). The endothelial cells were harvested until 80% confluence. Primary cultured aortic endothelial cells were verified using IF for CD31 (Figure 2(b)).

#### 2.6.3. WPMY-1 (ATCC® CRL­2854)

The normal human prostate stroma cell line WPMY-1 was purchased from the Cell Bank of the Chinese Academy of Sciences (Shanghai, China) and maintained in DMEM medium (HyClone, USA) with 10% fetal bovine serum (Gibco, USA).

### 2.7. RPrAE-RPrPF Coculture System and Transdifferentiation Experiments

The coculture system was established by a Transwell system (pore size 0.4 *μ*m, Corning, USA). RPrPF was seeded into the lower chambers of plates with the completed primary fibroblast medium, and RPrAE was added into transwells inserted in each well (pore size 0.4 *μ*m, Corning, USA). After 24 hours, the medium was changed to serum-free DMEM medium. Subsequently, as previously reported [16], cells were stimulated with either 1 ng/ml recombinant human TGF-*β*1 to induce transdifferentiation or 1 ng/ml recombinant rat bFGF to maintain the fibroblast phenotype. To study the effect of KLK1 on transdifferentiation, 5 nM recombinant human KLK1 and 10 nM LMWK were preincubated in the transwells for 30 min before TGF-*β*1 treatment in the lower chambers. Moreover, 100 nM HOE140 was used to study the antagonistic effect of BK II receptor, and it was preincubated in the transwells for 30 min before KLK1 and LMWK administration. As previously reported, stimulating the generation of intracellular cGMP by the soluble NO donor sodium nitroprusside (SNP) dose-dependently inhibited TGF-*β*1-induced transdifferentiation in the rat prostate fibroblasts [16]. For the positive control, 100 *μ*M SNP were preincubated for 30 min before TGF-*β*1 treatment. Administration was executed every 18 h from day 2 to day 3. Then, the mRNA and protein were extracted for test. All experiments were performed with cells from at least three individual donors.

### 2.8. Cell Proliferation Assays

Cell proliferation was determined using the CellTiter 96® AQ_ueous_ One Solution Cell Proliferation Assay kit (Promega, USA) according to the manufacturer's protocol as previously described [26, 27]. Briefly, the cells were seeded at 1000 cells/well into 96-well plate (Corning, USA). After adhesion, the complete media was substituted with appropriate basal media, and the cells were stimulated with the indicated concentrations of BK (R&D, USA) or vehicle. The plates were then incubated for 4 days, and cell growth was measured at daily intervals from the next day to the fifth day.

### 2.9. RNA Isolation and Quantitative Real-Time PCR (QRT-PCR)

Total RNA was extracted from frozen tissues and cell lines with the TRIzol reagent (Invitrogen, USA) and quantitated at 260/280 nm using a NanoDrop ND-1000 spectrophotometer (NanoDrop Technologies, USA). One *μ*g of each total RNA sample was reverse transcribed using a PrimeScript™ RT Master Mix (TaKaRa, China). QRT-PCR was performed to determine the mRNA levels of genes of interest based on SYBR reagent kit (TaKaRa, China) using a QuantStudio™ 6 Flex Real-Time PCR System (Thermo Fisher, USA). The mRNA expression levels of the examined genes were normalized to that of *β*-actin; the relative mRNA expression levels were calculated using the 2^−ΔΔCt^ method. All samples were independently repeated for analysis three times.

### 2.10. Western Blot Analysis

Total tissues and cellular proteins were extracted and separated by 10% sodium dodecyl sulfate polyacrylamide gel electrophoresis and then transferred onto polyvinylidene fluoride membranes (Millipore, USA). The membranes incubated overnight at 4°C with primary antibodies and then incubated with horseradish peroxidase-conjugated secondary antibodies for 1 h. After washing three times with Tris-buffered saline with Tween-20, the bands were analyzed using an enhanced chemiluminescence detection system (Pierce; Thermo Fisher Scientific). The data were normalized using *β*-actin as an internal control. All samples were analyzed independently via three repetitions, and the mean values were determined.

### 2.11. Histological Examinations

#### 2.11.1. Hematoxylin-Eosin (H&E) and Masson's Trichrome Staining

The 4% paraformaldehyde-fixed prostate tissue samples were subjected to paraffin embedding and sectioned at a thickness of 5 *μ*m. The tissue sections were stained with H&E Staining Kit (Solarbio, China) and Masson's Trichrome Stain Kit (Solarbio, China) using standard procedures, respectively, and imaged under a light microscope (Olympus, Japan). Smooth muscle (red) and collagen contents (blue) in the prostate were semiquantitatively analyzed using ImagePro Plus version 6.0. A total of 10 fields at 100x magnification were examined for each histologic slice.

#### 2.11.2. Immunohistochemistry (IHC)

Sections were incubated overnight at 4°C with antibodies against KLK1. Sections were then washed three times and incubated with a biotinylated secondary antibody. Finally, antigen-antibody reactions were detected by staining with diaminobenzidine (Beyotime, China). As above, semiquantitative analysis was performed to evaluate average optical density (AOD) of KLK1 positive area using ImagePro Plus.

#### 2.11.3. Immunofluorescence

Cells were cultured on glass coverslips pretreated with TC (Solarbio, China) in six-well plates and when the intervention was finished, they were fixed with 4% paraformaldehyde at room temperature for 15 min. The slides were then blocked and incubated overnight with the primary antibodies against E-Cadherin, vimentin, *α*-SMA, CD31, and B2 bradykinin receptor (BDKRB2). After incubation with the secondary antibody, DAPI (Southern Biotech, USA) was used for staining the nucleus. Stained cells were viewed by a fluorescence microscope (Olympus, Japan).

### 2.12. Assessment of NO, cGMP, cAMP, ADMA, Malondialdehyde (MDA), and Superoxide Dismutase (SOD)

These indicators of rat prostate tissues or cells were detected using commercial kits according to the manufacturer's instructions. The ADMA ELISA kit (Catalog No. RA20756) was purchased from the Bioswamp Life Science Lab (China). The cAMP ELISA kit (Catalog No MB-6668A) and cGMP ELISA kit (Catalog No. MB-1568A) were purchased from the Jiangsu Meibiao Biological Technology (China). The NO detection kit (Catalog No. A013-2-1), MDA detection kit (Catalog No. A003-1-2), and SOD detection kit (Catalog No. A001-3-2) were purchased from the Nanjing Jiancheng Bioengineering Institute (China). The assays were performed in triplicate, and the total protein concentrations were detected to normalize the data.

### 2.13. Substances

In Western blot experiments, the primary antibodies against rat KLK1 (Catalog No. ab131029) and human KLK1 (Catalog No. ab28289) were purchased from Abcam (USA); the primary antibodies against NADPH oxidase 2 (NOX2) (Catalog No. 19013-1-AP), NOX4 (Catalog No. 14347-1-AP), p47^phox^ (Catalog No. 28187-1-AP), p67^phox^ (Catalog No. 15551-1-AP), Collagen I (Catalog No. 14695-1-AP), Collagen III (Catalog No. 22734-1-AP), *α*-SMA (Catalog No. 14395-1-AP), DDAH2 (Catalog No. 14966-1-AP), TGF-*β*1 (Catalog No. 21898-1-AP), RhoA (Catalog No. 10749-1-AP), ROCK1 (Catalog No. 21850-1-AP), COX-2 (Catalog No. 12375-1-AP), and *β*-actin (Catalog No. 20536-1-AP) were purchased from Proteintech (China); the primary antibodies against PTGIS (Catalog No. DF4745), inducible NO synthase (iNOS) (Catalog No. AF0199), eNOS (Catalog No. AF0096), *β*-Tubulin (Catalog No. T0023), and the secondary antibodies were from Affinity (USA). In immunohistochemistry experiments, the KLK1 antibody (Catalog No. 32443) reactive to both human KLK1 and rat KLK1 was purchased from Signalway Antibody (USA); the secondary antibody was purchased from Proteintech (China). In the immunofluorescence experiments, the primary antibodies against E-Cadherin (Catalog No. 20874-1-AP), vimentin (Catalog No. 10366-1-AP), and *α*-SMA (Catalog No. 14395-1-AP) were purchased from Proteintech (China); the antibody against CD31 (Catalog No. AF6191) was purchased from Affinity (USA); and the antibody against BDKRB2 (Catalog No. bs-2422R) was purchased from Bioss (China); the secondary antibody was purchased from Thermo Fisher Scientific (USA). In the *in vitro* experiments, the recombinant human TGF-*β*1 (Catalog No. 100-21) and recombinant rat bFGF (Catalog No. 400-29) were purchased from PeproTech (USA); the recombinant human KLK1 (Catalog No. AP70473) was purchased from Signalway Antibody (USA); the LMWK (Catalog No. K3628) was purchased from Sigma (USA); the HOE140 (Catalog No. 3014) was purchased from R&D (USA); and the SNP (Catalog No. S9560) was purchased from Solarbio (China).

### 2.14. Statistical Analysis

Data were analyzed using SPSS version 21.0 (IBM, USA). Continuous variables are expressed as mean ± standard deviation (SD). Data were analyzed using one-way analysis of variance followed by Tukey's multiple comparison test to identify differences among multiple groups. Correlations of the 3 variables (age, the ratio of Masson positive area, KLK1 AOD) were analyzed by the Spearman correlation analyses. Statistical differences were regarded significant when *P* < 0.05.

## 3. Results

### 3.1. Verification of TGR

**Figure 3:**
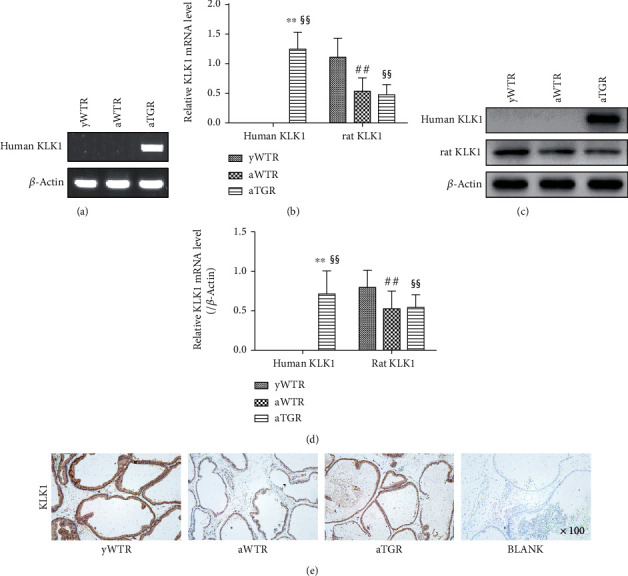
Verification of the existence and expression of human KLK1 and rat KLK1 genes in the prostate. (a) Representative human KLK1 genomic DNA bands in the prostate through conventional PCR followed by agarose gel electrophoresis. (b) Relative mRNA expression of the human KLK1 and rat KLK1 to *β*-actin within the prostate of different groups by RT-PCR. (c, d) Rat KLK1 and human KLK1 protein expression in the prostate by Western blot. (e) KLK1 protein expression and location in the prostate by IHC (the KLK1 antibody used in this experiment is reactive to both human KLK1 and rat KLK1; magnification ×100). For each group, values are presented as the mean ± SD of 10 rats per group. ^∗∗^*P* < 0.01 (aTGR vs. aWTR). ^##^*P* < 0.01 (aWTR vs. yWTR). ^§§^*P* < 0.01 (aTGR vs. yWTR).

The presence and expression of human KLK1 gene in rat prostate samples were verified at the levels of genomic DNA, mRNA, and protein (Figures 3(a)–3(c)). The results showed that rats in only the aTGR group contained the human KLK1 gene (Figures 3(a)–3(c)). In addition, rat KLK1 expression was lower in the two aged groups than that in the young group at both mRNA and protein levels (all *P* < 0.01, Figures 3(b)–3(d)).  Figure 3(e) showed the actual expression of KLK1 in the prostate of each group of rats by IHC (the primary antibody used in the IHC experiment could react to both human KLK1 and rat KLK1).

### 3.2. The Prostate KLK1 Gene Expression Was Downregulated in Elderly Man

**Figure 4:**
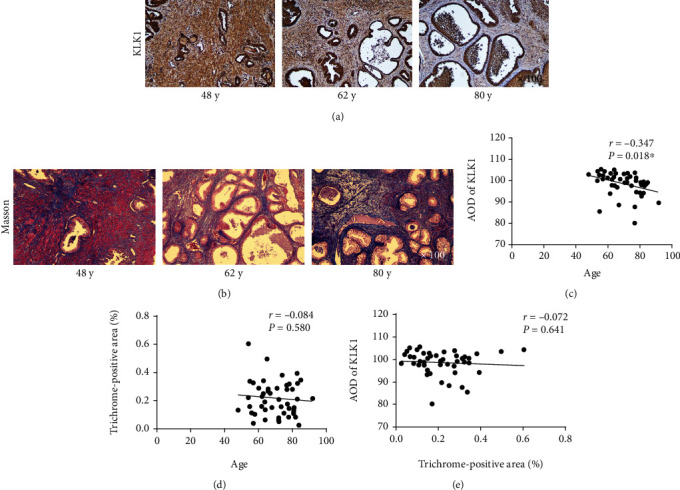
Expression of KLK1 in prostate surgical specimens of patients with BPH and correlation among KLK1, age, and prostate fibrosis. (a, b) Representative IHC photos of KLK1 and Masson photos in different ages of BPH patients, respectively (magnification ×100). (c–e) Pairwise correlations between KLK1 AOD, age, and the area ratio of collagen (*n* = 47), respectively.

We performed IHC to observe KLK1 expression in prostates of BPH patients whose age from 48 to 92 years. Figures 4(a) and 4(b) showed representative images of KLK1 IHC and Masson staining for prostates of different ages. Spearman rank correlation analyses showed that the AOD of KLK1 was significantly associated with age (*r* = −0.347, *P* = 0.018, Figure 4(c)). Masson's trichrome staining was also performed among these samples. This suggested that the expression of KLK1 decreases with age in human prostate. However, there was no significant correlation between the area ratio of collagen and age (*r* = −0.084, *P* = 0.580, Figure 4(d)) or the AOD of KLK1 (*r* = −0.072, *P* = 0.641, Figure 4(e)).

This unexpected result suggested that neither age nor KLK1 might be the most important cause of fibrosis. But it should be noted that BPH is a multifactorial disease, and inflammation might be the most unpredictable factor. Inflammation can induce the upregulation of KLK1 which exerts antifibrosis effect [28, 29], but at the same time, inflammation can also lead to the secretion of other profibrosis factors [30]. The balance between profibrosis and antifibrosis has different tendencies in different individuals, and inflammation may occur randomly in any periods of BPH. Therefore, it was difficult to bring the true relationship between KLK1, age, and fibrosis to the surface from our data.

### 3.3. KLK1 Suppressed Fibrosis and Oxidative Stress in the Prostate of Aged Rat

**Figure 5:**
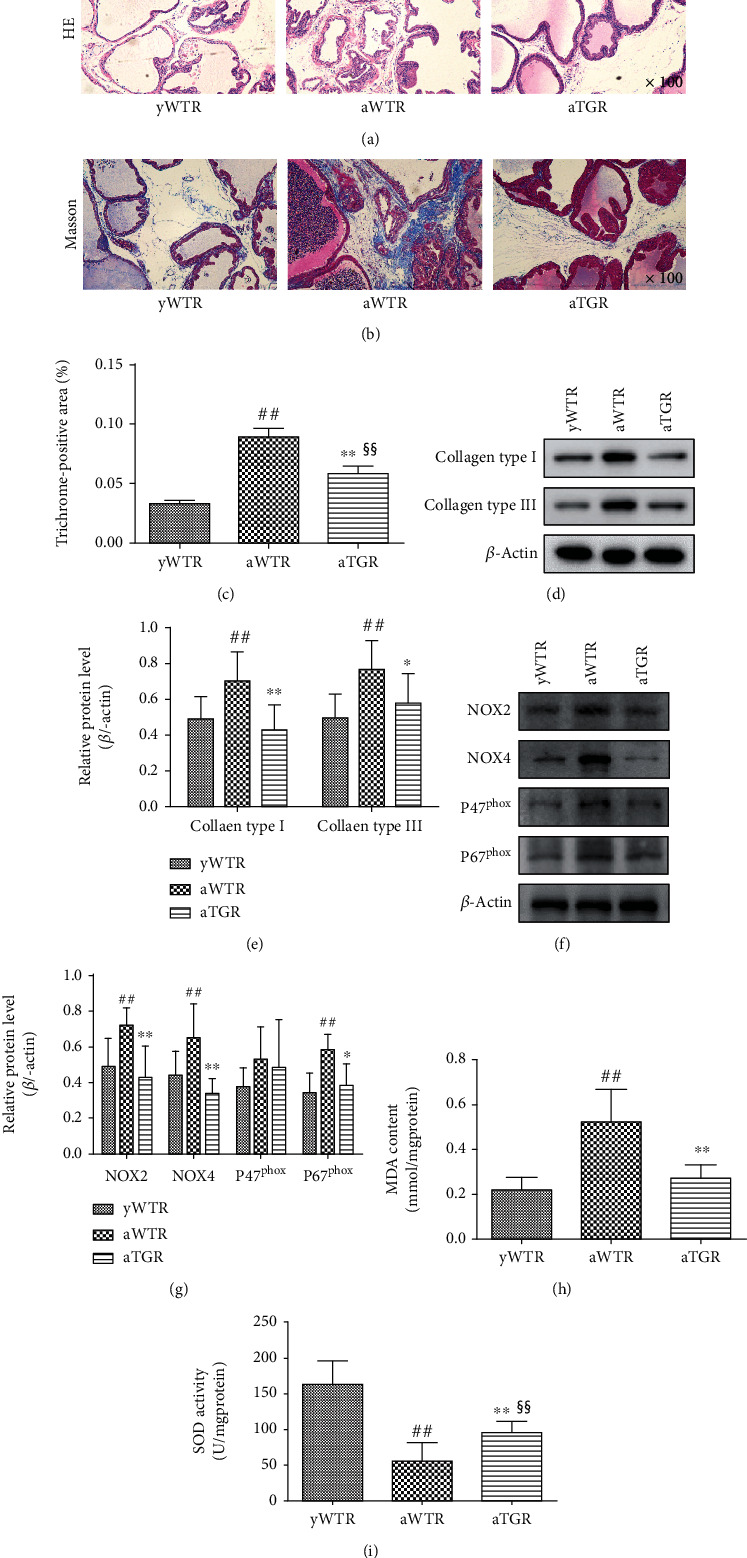
Pathological changes and oxidative status of prostate in aged wild rats and aged human KLK1 transgenic rats. (a, b) Representative HE photos and Masson photos in rat prostates (magnification ×100). (c) The area ratio of collagen in the Masson photos of rat prostates. (d, f) Representative Western blot results of Collagen I, Collagen III, NOX2, NOX4, p47^phox^, and p67^phox^ in prostates of all three groups. (e, g) The expression levels of above-mentioned proteins with *β*-actin as the loading control in all three groups. (h, i) MDA content and SOD activity normalized to total protein concentration of rat prostate samples. For each group, values are presented as the mean ± SD of 10 rats per group. ^∗^*P* < 0.05, ^∗∗^*P* < 0.01 (aTGR vs. aWTR). ^##^*P* < 0.01 (aWTR vs. yWTR). ^§§^*P* < 0.01 (aTGR vs. yWTR).

The results of HE staining showed that the prostate of aTGR had “younger” manifestations, such as a fuller epithelium, a more regular glandular cavity, and a looser stroma (Figure 5(a)). By Masson's trichrome staining, the area ratios of collagen were lower in the aTGR group than that in the aWTR group (*P* < 0.01, Figures 5(b) and 5(c)), indicating less fibrosis. Results of Western blot analysis, consistent with the pathological results, showed expression levels of Collagen I and Collagen III were lower in the yWTR and aTGR groups compared with the aWTR group (all *P* < 0.05, Figures 5(d) and 5(e)).

Members of the NOX family are the main source of ROS. P47^phox^ and p67^phox^ are cytosolic regulatory subunits of NOX [31]. Compared to the yWTR and aTGR group, the protein expression of NOX2, NOX4, and p67^phox^ of the aWTR group increased significantly (all *P* < 0.05, Figures 5(f) and 5(g)), while the aTGR group showed no significantly difference when compared to the yWTR group (*P* > 0.05). The expressions of p47^phox^ had no difference between the WTR and TGR groups (*P* > 0.05). MDA as an indicator for oxidative damage showed a similar trend as NOX2 and NOX4 (*P* < 0.01, Figure 5(h)). Contrarily, SOD, an antioxidant, presented reverse activity changes as MDA (*P* < 0.01, Figure 5(i)).

### 3.4. The Mechanism Involved in KLK1's Effect on Attenuating Aging-Related Changes in Rodent Prostates

**Figure 6:**
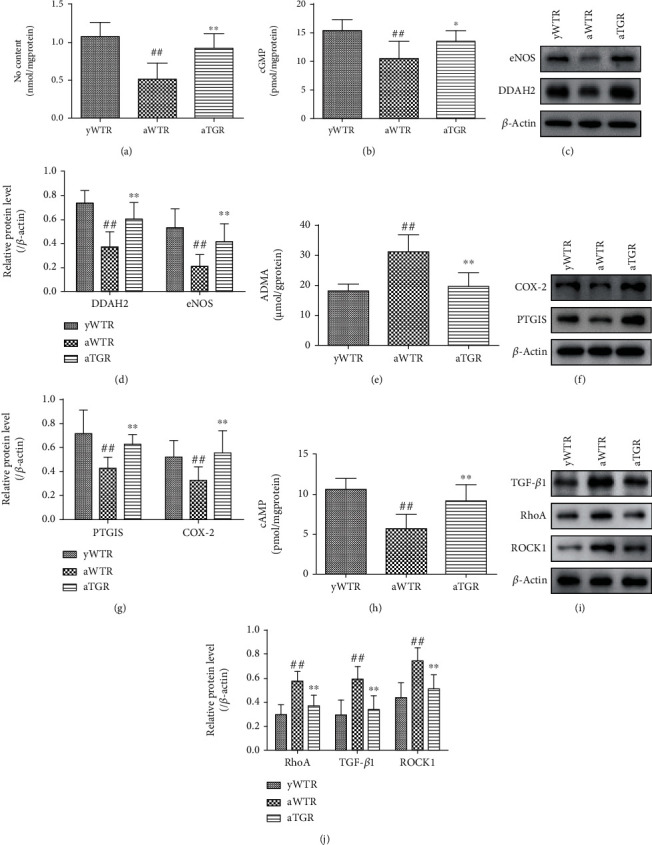
KLK1 could upregulate NO/cGMP signal and inhibit fibrosis in the prostate of aged rats. (a, b, e, and h) NO content, cGMP content, ADMA content, and cAMP content normalized to total protein concentration of rat prostate samples. (c, f, and i) Representative Western blot results of eNOS, DDAH2, COX-2, PTGIS, TGF-*β*1, RhoA, and ROCK1 in prostates of all three groups. (d, g, and j) The expression levels of above-mentioned proteins with *β*-actin as the loading control in all three groups. For each group, values are presented as the mean ± SD of 10 rats per group. ^∗^*P* < 0.05, ^∗∗^*P* < 0.01 (aTGR vs. aWTR). ^##^*P* < 0.01 (aWTR vs. yWTR).

Our experimental results suggested that human KLK1 upregulated NO and cGMP in the prostate of aged TGRs via DDAH/ADMA/eNOS pathway. Significant increase in NO and cGMP was found in the aTGR group compared to the aWTR group (both *P* < 0.05, Figures 6(a) and 6(b)). Then, we investigated the reasons for the increase of NO. Compared to the aWTR group, Western blot results showed a significant increase in eNOS and DDAH2 protein levels in the aTGR group (both *P* < 0.01, Figures 6(c) and 6(d)), and ELISA results showed a significant decrease in ADMA in the aTGR group (*P* < 0.01, Figure 6(e)). Our previous research found that KLK1 upregulates NO by increasing DDAH and NOS in corpus cavernosum [18], and this time, the effect in the prostate has been also proved.

Human KLK1 also activated the COX-2/PTGIS/cAMP pathway in the prostate of aged TGRs. Results of Western blot showed a significant decrease in COX-2 and PTGIS protein levels in the aWTR group compared to the yWTR group, while the human KLK1 restored the protein expression of COX-2 and PTGIS in the aged transgenic rats (both *P* < 0.01, Figures 6(f) and 6(g)). Consistently, based on ELISA results, the downstream cAMP was also significantly increased by KLK1 in the aTGR group compared to the aWTR group (*P* < 0.01, Figure 6(h)).

The TGF-*β*1/RhoA/ROCK1 pathway in the prostate of aged TGRs was suppressed. Western blot analysis results showed that expressions of TGF-*β*1, RhoA, and ROCK1 in aWTR group were higher compared with groups of yWTR and aTGR (all *P* < 0.01, Figures 6(i) and 6(j)), indicating that human KLK1 blocked the aging-related fibrosis in the aged transgenic rats through the RhoA/ROCK1 pathway.

### 3.5. KLK1 Upregulates NO in RPrAE and Inhibited TGF-*β*1-Mediated Fibroblast-to-Myofibroblast Transdifferentiation in the RPrAE-RPrPF Coculture System

We verified RPrPF and RPrAE using IF for specific markers. According to cell morphology and the expression of specific markers (Figures 2(a) and 2(b)), we came to the conclusion that RPrPF and RPrAE were successfully isolated and passaged.

The activation of the RhoA/ROCK1 pathway upregulated by TGF-*β*1 promotes cytoskeletal rearrangement and initiates the fibroblast-to-myofibroblast transdifferentiation [5, 7]. The RPrPF was observed to maintain a fibroblast phenotype in medium with bFGF, as evidenced by expression of vimentin filaments and lack of *α*-SMA (Figures 2(a) and 2(c)). In contrast, TGF-*β*1 treatment induced transdifferentiation of RPrPF, marked by high percentage of the cells stained positive for *α*-SMA (Figure 2(c)). In RPrAE, direct administration of BK (10 nM) or simultaneous administration of KLK1 (5 nM) and LMWK (10 nM) could upregulate eNOS expression and NO production (both *P* < 0.05, Figures 2(d)–2(f)). In the coculture system, through the production of NO, RPrAE mediated the effect of KKS on RPrPF transdifferentiation. In the presence of RPrAE and LMWK, transdifferentiation was significantly attenuated by KLK1 as determined by the reduction of *α*-SMA (*P* < 0.01, Figures 2(g)–2(i)), while HOE140 administration abolished this effect (*P* < 0.01). The positive control group with SNP 1treatment also exhibited repressed transdifferentiation, which indicated that KLK1 exerted its effect by causing NO production in the endothelium.

### 3.6. The Direct Effects of BK on Prostate Cells

**Figure 7:**
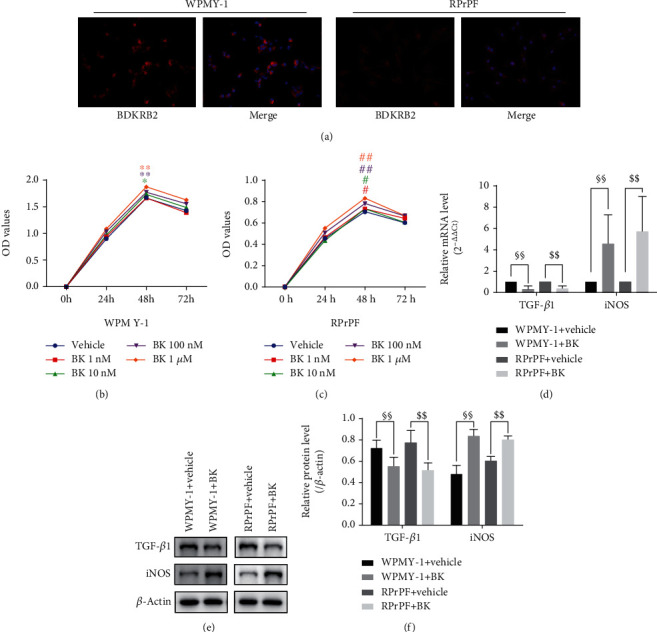
The direct effects of BK on prostate cells. (a) The BDKRB2 (red) expression in WPMY-1 and RPrPF (magnification ×200). (b, c) MTT results of WPMY-1 and RPrPF under administration of BK in different concentrations. Results were plotted as OD values. ^∗^*P* < 0.05, ^∗∗^*P* < 0.01 (WPMY-1 + BK vs. the vehicle group at the same time point in 3 independent repeated experiment). ^#^*P* < 0.05, ^##^*P* < 0.01 (RPrPF + BK vs. the vehicle group at the same time point in 3 independent repeated experiment). (d) Relative mRNA expression of the TGF-*β*1 and iNOS to *β*-actin within WPMY-1 and RPrPF under 10 nM BK by RT-PCR. (e, f) TGF-*β*1 and iNOS protein expression within WPMY-1 and RPrPF under 10 nM BK by Western blot. Each bar represents mean ± SD of 3 independent repeated experiment. ^§§^*P* < 0.01 (WPMY-1 + BK vs. WPMY-1 + vehicle). ^$$^*P* < 0.01 (RPrPF + BK vs. RPrPF + vehicle).

The indirect effects of BK on prostate fibroblast had been studied via the RPrAE-RPrPF coculture system. However, considering that prostate stromal cells expressed B2 bradykinin receptors [32], it was necessary to study the direct effects of BK on them.  Figure 7(a) showed that WPMY-1 and RPrPF expressed BDKRB2 by IF. The WPMY-1 and RPrPF cell proliferation ability was significantly upregulated after BK administration (*P* < 0.01, Figures 7(b) and 7(c)). But within the range of BK concentration (1 nM, 10 nM, 100 nM, and 1 *μ*M) used in our experiments, the increases in cell proliferation were quite low. In addition, tested by Western blot and QRT-PCR, 10 nM BK upregulated iNOS and inhibited TGF-*β*1 expression in both WPMY-1 and RPrPF (*P* < 0.01, Figures 7(d)–7(f)). This preliminarily suggested that BK may be beneficial to prevent the progression of fibrosis via the direct effect on prostatic stroma cells. However, the unanticipated role that may accompany it remains to be studied.

## 4. Discussion

Our study is the first to describe a protective role of KLK1 on BPH. Limited to the study, our data suggested that with the age increasing, the expression of KLK1 decreased in the prostate in both human and rat. Harboring the human KLK1 gene could weaken the oxidative stress and fibrosis in aged rat prostate by regulating the DDAH/ADMA/eNOS/NO/cGMP pathway and the COX-2/PTGIS/cAMP pathway, as well as suppress the TGF-*β*1/RhoA/ROCK1 pathway to inhibit fibroblast-to-myofibroblast transdifferentiation which had also been verified in a cocultivation system *in vitro*.

During late life, the accumulation of senescent fibroblasts which has altered transcriptome is blamed for stromal changes in BPH [33]. Senescent fibroblasts secrete higher levels of extracellular matrix (ECM) proteins and inflammatory cytokines, leading to chronic inflammatory and tissue repair responses [33]. Notably, TGF-*β*1, an essential profibrotic cytokine, upregulated in many pathological occasions like chronic inflammation and old age, strongly induces myofibroblast differentiation in prostate [34]. Due to its capability of combining the ECM-producing characteristic of fibroblast and contractile property of smooth muscle cells, myofibroblast plays a central role during normal wound healing, whereas excessive activation and abortive apoptosis of myofibroblast would result in persistent myofibroblast activation, cellular proliferation, and ECM deposition [5]. Moreover, the accumulated ECM will reduce the degradation of certain growth factors and even promote their effects [5]. Therefore, the key to ameliorate aging-related BPH is to block the excessive transdifferentiation of fibroblast-to-myofibroblast and maintain the ECM homeostasis.

However, TGF-*β*1 may not be considered a direct clinical target due to its critical function in diverse biological processes and homeostatic maintenance [35]. Sampson et al. reviewed that myofibroblast differentiation induced by TGF-*β*1 was driven by a prooxidant shift in redox homeostasis due to elevated production of NOX4-derived hydrogen peroxide and supported by concomitant decreases in NO/cGMP signaling and ROS scavenging enzymes [5]. Fujii et al. reported that NO inactivated NOX by inhibiting its assembling process [36]. Zenzmaier et al. found that amplified NO/cGMP pathway reduced the proliferation of fibroblasts and TGF-*β*1-induced myofibroblast differentiation [16]. Thus, the NO/cGMP pathway is believed to be a better target for attenuating prostate oxidative stress and fibrosis. Encouragingly, in our experiments, activation of the KKS not only promoted the NO/cGMP pathway but also reduced the levels of TGF-*β*1 and NOX4.

The kallikrein/kinin-related protection effects are mainly mediated by a B2 bradykinin receptor-NO-dependent event that BK binds the membrane-bound B2 receptor with downstream phosphorylation of eNOS and production of NO [37]. Over the past two decades, many studies have reported the inhibitory effect of KLK1 on TGF-*β*1. Yin et al. reported KLK1 prevented inflammation and limited ventricular remodeling after myocardial ischemia/reperfusion by suppressed oxidative stress, TGF-*β*1/Smad pathway, and NF-*κ*B activation [38]. Cardenas et al. reported upregulation of the B2 bradykinin receptor pathway could modulate the TGF-*β*1/Smad signaling cascade to reduce renal fibrosis induced by bovine serum albumin [39]. Our previous work verified that KLK1 ameliorated corporal fibrosis via suppressing TGF-*β*1 and RhoA/ROCK1 pathway in aging-related erectile dysfunction [21]. In this study, we also found that KLK1 (1) increased NO production by keeping eNOS activity and (2) inhibited ECM deposition by increasing prostaglandin secretion, and then, NO suppressed oxidative stress, relaxed prostate smooth muscle, and reduced the fibrotic factors in the prostate. Furthermore, we demonstrated that cocultured with the endothelial cells, KLK1 inhibited fibroblast-to-myofibroblast transdifferentiation mediated by TGF-*β*1 *in vitro*. Therefore, it was sufficient to believe that KKS, represented by KLK1, was a potential therapeutic target for BPH. However, one of the shortcomings in our animal experiments is the omission of the young TGRs. Therefore, we should state that our conclusions were limited to this research, and more complete and rigorous experiments should be carried out when conditions permit.

Since BK is a well-known inducer of the proinflammatory response, it is necessary to study its direct effects on prostate cells. Srinivasan et al. reported that 10 nM BK caused a cellular proliferation of normal prostate stromal cells, but not of the normal prostate epithelial or prostate cancer cell lines [32]. Similar to their observation, we also found 10 nM BK promoted proliferation of WPMY-1 and primary prostate stromal cells, but the increase is quite low compared to the control group. On the other hand, although it has been shown that activation of the KKS system promoted the production of NO through the vascular endothelium and relaxed prostate smooth muscle, BK has also been reported as a substance that contracts the prostate smooth muscle. Watts et al. found that BK (10-1000 nM) was an effective contractile agonist in an isolated ventral lobe of the rat's prostate [40]. Srinivasan et al. also reported that activation of the B2 bradykinin receptor by BK produced a concentration-dependent mobilization of intracellular calcium in human prostate stromal cells, and a significant difference can be observed at a concentration of 10 nM [32]. However, it is worth noting that BK is extremely easily degraded *in vivo*. Kinins are degraded by enzymes such as neutral endopeptidase, kininase I, and kininase II which is also known as angiotensin-converting enzyme (ACE). Nassis et al. reported that ACE was localized to glandular epithelial cells in the human prostate with a significant increase in ACE expression in BPH compared with the normal prostate [41]. Chao indicated that kinins have a short half-life of approximately 30 s; it is unlikely that tissue kallikrein administration can cause excessive kinin production [13]. Therefore, we believed that at least in our experiments, BK had a beneficial effect on the prostate by promoting the production of NO via vascular endothelium, and because of its rapid degradation, BK did not obviously cause the proliferation or contraction of rat prostate stromal cells *in vivo*. Additionally, Naidu et al. indicated that BK could mediate metastasis and invasion of the prostate cancer cells, even associated with angiogenesis [42]. However, in benign cases, the benefits and disadvantages of KKS for the prostate are worthy of discussion. In our current study, human KLK1 transgenic rats naturally grew to old age without any malignant manifestations in their prostates. So it could be speculated that KLK1 was not a predisposing factor for prostate cancer.

KLK1, by activating KKS, has played many beneficial effects. Moreover, Kailikang, a drug made from human urine KLK1, has been widely applied to treat human acute ischemic stroke clinically in China [43]. Through this study on aged human KLK1 TGRs and surgical specimens of BPH patients, we have reason to believe that activating KKS and subsequent signaling pathways by KLK1 have a very beneficial effect on human BPH, but this also requires more complete and in-depth experiments to study. In this article, our study was mainly based on rats with natural aging. However, we did not discuss and explore the sex hormone imbalance, which is another recognized mechanism of BPH development [44]. The latest research reported that old rats were more susceptible to induction of BPH at comparative to young mature probably determined by a higher expression of androgen receptors in old animals [45]. In order to apply KLK1 to BPH patients in the future, it is necessary to study the interaction between KLK1 and sex hormones and sex hormone receptors. On the other hand, inflammation is one of the important mechanisms of BPH progression discovered in recent years [30, 46], and KLK1 exhibits anti-inflammatory effects in some acute ischemic injuries [13]. Therefore, it is also necessary to ascertain the influence of KLK1 on the inflammatory mechanism of BPH. We recently studied the relationship between chronic inflammation and BPH through the experimental autoimmune prostatitis modeling method, and it was found that the expression of a variety of cytokines which promote the proliferation of stroma components increased in the prostate of inflammation model group [47]. Our next experimental plan is to use KLK1 administration on the testosterone-induced BPH rat model and chronic prostatitis rat model to study the interaction of KLK1 on hormones and inflammation-related mechanisms.

## 5. Conclusions

We concluded that KLK1 played a protective role in aging-related changes in rodent prostates and was downregulated in elderly prostate in both human and rat. The DDAH/ADMA/eNOS/NO/cGMP pathway and the COX-2/PTGIS/cAMP pathway were linked to the mechanism that KLK1 inhibit oxidative stress and reduce prostate fibrosis. And we also preliminarily explored the potential adverse effects of KLK1 on the prostate, which might not be serious.

In particular, through surgical specimens obtained clinically from patients with BPH, we found that the expression of KLK1 in the prostate gradually decreases with age (determined by IHC), which provides important clues for the subsequent use of KLK1 and related mechanisms for the treatment of human BPH.

## Data Availability

The data of the materials and methods and results to support the conclusions are included in this article. If any other data are needed, please contact the corresponding author.
